# Neutral theory: applicability and neutrality of using generic health-related quality of life tools in diseases or conditions where specific tools are available

**DOI:** 10.1186/s12874-021-01279-w

**Published:** 2021-04-26

**Authors:** Ravi Jandhyala

**Affiliations:** 1 Medialis Limited, Banbury, UK; 2 Centre for Pharmaceutical Medicine Research, Institute of Pharmaceutical Science, Faculty of Life Science & Medicine, King’s College London, London, UK

**Keywords:** SF-36, EQ-5D, Utility, Patient-reported outcomes (PRO), Health-related quality of life, Construct, Neutral theory

## Abstract

**Background:**

Health-related quality of life (HRQoL) tools are limited by the indicators included in the construct and variation in interpretation by different researchers. Neutral Theory describes the ideal construct that includes all relevant indicators and, therefore, complete accuracy, or neutrality. Neutral Theory can thereby provide the framework to develop or test constructs. To assess the application of Neutral Theory, the neutrality of generic tools (SF-36 and EQ-5D) at measuring HRQoL was compared to disease/condition-specific tools, with the latter considered surrogates for the Neutral construct.

**Methods:**

Full descriptions of all disease/condition-specific HRQoL tools published on PubMed (to 01-Jul-19) were sourced. For each tool, the number of items with and without a direct match within the SF-36 and EQ-5D was recorded and the sensitivity/specificity calculated.

**Results:**

The SF-36 and EQ-5D did not achieve a sensitivity/specificity both > 50% against any of the 163 disease/condition-specific tools identified. At 20% prevalence of poor HRQoL, the false positive rate (FPR) was > 75% for all but two tools against the SF-36 and six tools against the EQ-5D. Increasing poor HRQoL to 80%, 47 tools for the SF-36 and 48 tools for the EQ-5D had a FPR < 50%. For rare disease tools (< 1/2000 population; *n* = 17), sensitivity/specificity ranged from 0 to 40%/5–31% for the SF-36 and 0–22%/29–100% for the EQ-5D. For non-rare (*n* = 75) and symptom-specific tools (*n* = 71) sensitivity/specificity was: 0–100%/0–100% (SF-36) and 0–50%/0–100% (EQ-5D); and 0–60%/0–19% (SF-36) and 0–25%/0–100% (EQ-5D), respectively. No concordance was recorded for 18% (2/11) of results from studies of rare disease tools versus the SF-36 (no data vs EQ-5D). For non-rare, disease-specific tools, results were discordant for 30% (25/84) and 35% (23/65) of studies against the SF-36 and EQ-5D, respectively. For symptom-specific tools, corresponding results were 36% (24/66) and 16% (5/31).

**Conclusions:**

Generic HRQoL tools appear poorly correlated with disease/condition-specific tools, which indicates that adoption of Neutral Theory in the development and assessment of HRQoL tools could improve their relevance, accuracy, and utility in economic evaluations of health interventions.

**Supplementary Information:**

The online version contains supplementary material available at 10.1186/s12874-021-01279-w.

## Background

Observation of a construct, such as a quality of life tool, first requires conceptualisation of the construct at a theoretical level followed by its operationalisation at an empirical level. Operationalisation involves selecting indicators to be measured in the observation of the construct. Both are vulnerable to variation in interpretation by different researchers and can result in a divergence in their measurement of the ‘same’ construct. A new theory recently proposed is that of a ‘Neutral Observer’, which provides a framework on which a determination of the neutrality, or accuracy, of an observation of a given construct can be based [1]. Neutral Theory represents the ideal and assumes a Neutral or exhaustive list of relevant indicators in the construct, whereby the sensitivity and specificity are both 1 (i.e.*,* 100% accurate). The operationalisation of constructs using disease-specific indicators can perhaps be considered closer to achieving neutrality than those based on generic observations.

Understanding the impact of treatment on patients’ quality of life is a pivotal component in the economic evaluation of health interventions. There is, however, no universally agreed definition of the construct of quality of life, with the one provided by the World Health Organization (WHO) perhaps the most commonly cited: “an individual’s perception of their position in life in the context of the culture and value systems in which they live and in relation to their goals, expectations, standards and concerns” [2]. This broad definition includes the person’s physical health, psychological state, personal beliefs, social relationships and their relationship to salient features of their environment. The WHO definition, and other similar ones, were influential in the concept of health-related quality of life (HRQoL), which refers to how well a person functions in their life and his or her perceived well-being in physical, mental, and social domains of health [3].

Two independently operationalised tools that are frequently used to objectively assess HRQoL are: the Medical Outcomes Study Short Form family of measures (e.g. SF-36 [4, 5]) and the EuroQol five-dimensional (EQ-5D) [6, 7]. Both of these tools capture HRQoL (or, strictly speaking, health status for the EQ-5D [6, 8]) across a series of domains or dimensions: vitality, physical functioning, bodily pain, general health perceptions, physical functioning, emotional functioning, social functioning, and mental health in the SF-36 [5]; and mobility, self-care, usual activities, pain/discomfort, and anxiety/depression in the EQ-5D [7].

Generic HRQoL tools have been widely adopted in Health Technology Assessments (HTAs), with the National Institute for Health and Care Excellence (NICE) in the UK recommending use of the EQ-5D in its Technology Appraisals [9]. Generic HRQoL tools, by their nature, depict aspects of well-being and quality of life from the patients’ point of view across all diseases and, therefore, have utility in population-level studies as well as informing comparisons between diseases. While this allows for potentially more consistent, transparent and predictable decision-making, it is open to criticism, as generic measures may be insensitive or fail to capture important aspects of health for a specific disease or condition [10]. Disease- or condition-specific HRQoL tools have the advantage of being clinically relevant to the health problem and responsive to clinically important changes in state, such as the impact of treatment. Conversely, this specificity complicates comparisons with the general population and across treatments for different diseases, limiting their application in HTAs.

This study aimed to apply Neutral theory in assessing the neutrality, or accuracy, and applicability of generic tools (SF-36 and EQ-5D) at measuring HRQoL in diseases or conditions where there is a specific tool available, to act a surrogate for the Neutral list in the measurement of HRQoL.

## Methods

### Identification of disease- or condition-specific health-related quality of life tools

A literature search was performed to identify all published disease- and condition-specific HRQoL tools. Medline (PubMed) was searched through 01 July 2019 using the following terms: [“patient reported outcome” OR “PRO” OR “Quality of life” OR “QoL” AND “disease specific” OR “condition specific”]; limit: [English language]. Two reviewers undertook the search, with initial screening of abstracts and titles conducted using the semi-automated Rayyan tool (https://rayyan.qcri.org/) [11]. Full descriptions of the identified disease/condition-specific HRQoL tools were sourced as were the SF-36 and EQ-5D. In addition, all original studies where HRQoL was assessed using a disease/condition-specific HRQoL tool and the SF-36 and/or the EQ-5D were reviewed.

### Inclusion of appropriate domains and items

The risk that the generic tools (SF-36 and EQ-5D) might include irrelevant domains or items or exclude relevant domains or items for a specific disease or condition was assessed. Firstly, for each condition- or disease-specific tool the number of items with and without a direct match to the SF-36 and EQ-5D was recorded (for the EQ-5D, it was permitted for each of the five questions to cover more than one item in each disease/condition-specific tool). The sensitivity and specificity of the generic tool versus the disease/condition-specific tool was then calculated as follows. True positives represented items captured in both the disease/condition specific and generic tool; false positives, those captured in the generic tool, but not in the disease/condition specific tool; and false negatives, those captured in the disease/condition specific tool, but not in the generic tool. Since it is not possible to know if the disease/condition specific tool fully captures all relevant items or domains, the true negative fraction was assumed to be 0.9 (i.e. an arbitrary 10% missing). Sensitivity/specificity results were further stratified into rare diseases (defined as affecting < 1 in 2000 population) [12], non-rare diseases (≥1 in 2000 population), and symptom-specific tools (i.e. those that cover symptoms [e.g. urological symptoms; respiratory problems *etc*] that might be present in multiple diseases/conditions).

The potential for misclassification of patients’ HRQoL by a generic tool was expressed as the median proportion of false positives and false negatives (with 95% prediction intervals), based on 1000 studies, with prevalence of poor HRQoL set at 20, 50, and 80%.

### Concordance of quality of life scores

For each of the studies comparing a disease/condition-specific tool with the SF-36 and/or EQ-5D, a measure of concordance of the results was assigned. *No* (*none*) concordance was assigned if a significant impact on HRQoL was seen with the disease/condition specific tool, but no change or the opposite impact was seen with the generic tool (or vice versa); *Moderate* concordance if HRQoL impact was scored in the same direction with both tools, but was statistically significant with only one of them; and *Strong* concordance if the results were fully aligned (significant/non-significant impact in same direction). For studies that measured HRQoL changes over time, it was determined whether the concordance between the generic and disease/condition-specific tool varied at different time points. Results were split into rare diseases non-rare diseases, and symptom-specific tools.

All analyses were performed using R 3.6.0 (Revolutions Analytics) and Microsoft Excel 365 (Microsoft).

## Results

### Identification of disease- or condition-specific quality of life tools

**Fig. 1:**
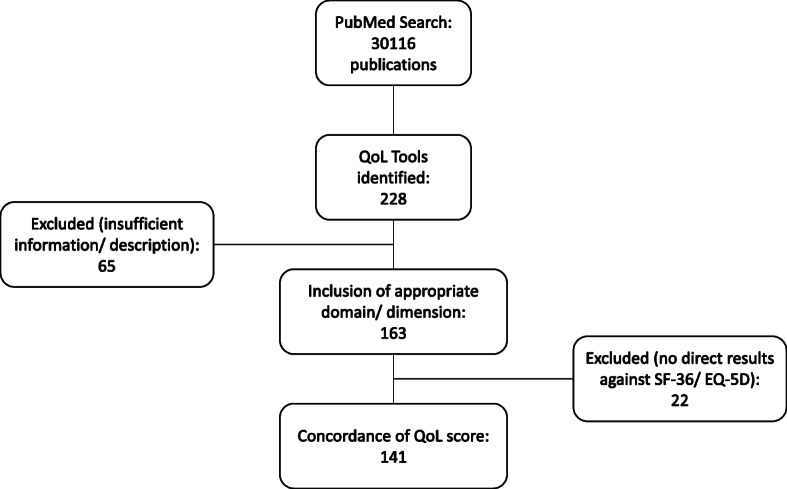
Overview of identification and selection of disease- and condition-specific health-related quality of life tools

A total of 30,116 publications were reviewed from which 228 discreet, disease- or condition-specific HRQoL tools were identified (Fig. 1 & Additional file 1). Full descriptions of 65 tools were unable to be sourced, either from published papers, online repositories, or via direct approaches to the authors. The remaining 163 tools (rare diseases: 17; non-rare: 75; symptom specific: 71) provided sufficient information/data for analysis, including 141 reporting results for a direct comparison against the SF-36 and/or EQ-5D (rare diseases: 10; non-rare: 73; symptom specific: 58). One tool (University of California Los Angeles-Prostate Cancer Index [UCLA-PCI]) completely overlaps with the SF-36, so was excluded from the SF-36 comparisons.

### Inclusion of appropriate domains and items

#### SF-36

The SF-36 had a sensitivity of > 75% against only one of the 162 disease/condition-specific HRQoL tools analysed, with a further five tools having a sensitivity between 50 and 75% (Additional file 2). A specificity of > 50% was achieved by the SF-36 against only one tool. The SF-36 did not achieve a sensitivity and specificity both > 50% against any of the 162 HRQoL tools. For the 17 rare disease HRQoL tools, sensitivity ranged between 0 and 40% and specificity between 5 and 31%. The corresponding rates for non-rare and symptom-specific tools were sensitivity: 0–100% and specificity: 0–100% and sensitivity: 0–60% and specificity: 0–19%, respectively.

**Fig. 2:**
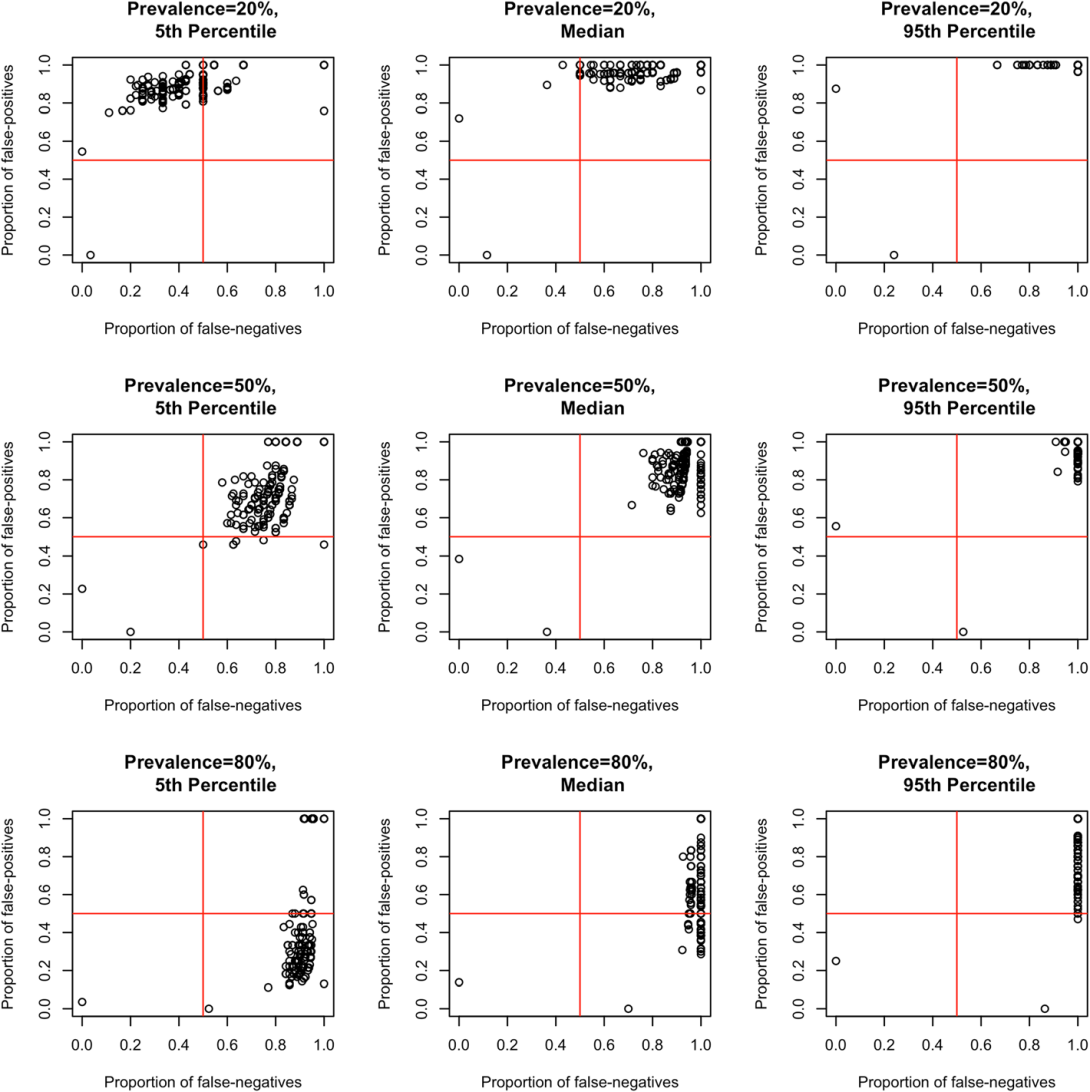
Misclassification (false positives and false negatives) of all disease/condition-specific tools against the SF-36. The figure presents misclassification of all quality of life tools (*n* = 163) against the SF-36 by nine panels: three prevalence values (rows: 20,50, 80%) and three points (columns: 5th percentile, median, 95th percentile of the prediction interval). Each point represents misclassification in 2 dimensions: proportions of false negatives (X-axis) and false positives (Y-axis)

At a prevalence of poor HRQoL of 50%, the proportion of false positives (FPR) was > 50% for 160/162 of the disease/condition-specific HRQoL tools and > 75% for 137/162 tools against the SF-36 (Fig. 2). The corresponding false negative rate (FNR) was > 50% for 160/162 tools and > 75% for 159/162 tools. Decreasing the prevalence of poor HRQoL to 20% increased the number of studies with a FPR of > 75% to 160/162 tools, while the FNR was < 50% for 12/162 tools. Conversely, increasing the prevalence of poor HRQoL to 80% resulted in 47/162 tools having a FPR < 50%, while 160/162 tools had a FNR > 75%.

#### EQ-5D

For the EQ-5D, a sensitivity of > 50% was not reached against any of the 163 disease/condition-specific HRQoL tools, with the highest recorded being 50% for one tool (Additional file 2). A specificity of > 50% for the EQ-5D was found against 27 HRQoL tools and > 75% against 13 tools. A sensitivity and specificity both > 50% was not achieved by the EQ-5D against any of the tools. Sensitivity and specificity ranged from 0 to 22% and 29–100%, respectively, against rare tools, 0–50% and 0–100% for non-rare tools, and 0–25% and 0–100% for symptom-specific tools.

**Fig. 3:**
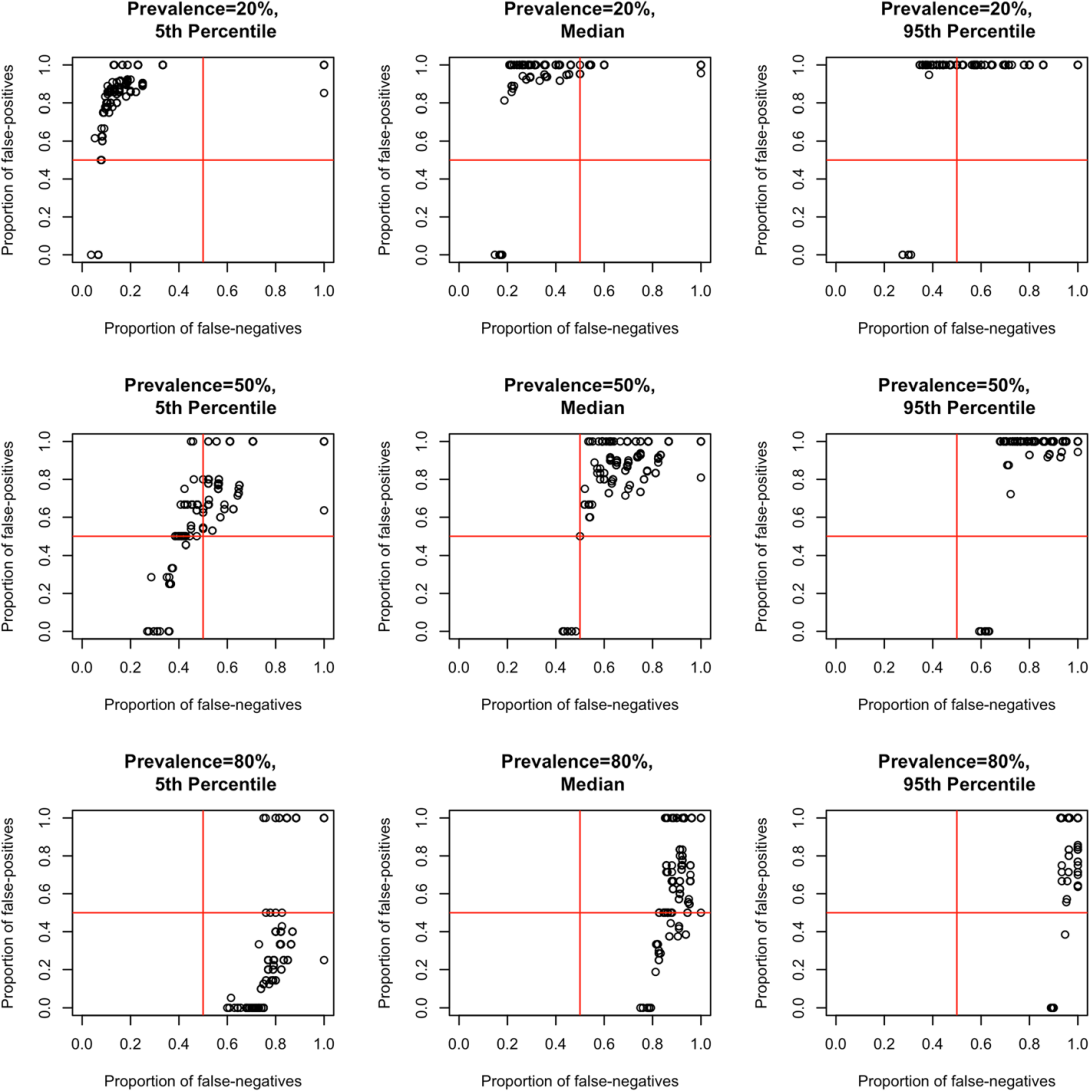
Misclassification (false positives and false negatives) of all disease/condition-specific tools against the EQ-5D. The figure presents misclassification of all tools (*n* = 163) against EQ-5D by nine panels: three prevalence values (rows: 20, 50, 80%) and three points (columns: 5th percentile, median, 95th percentile of the prediction interval). Each point represents misclassification in 2 dimensions: proportions of false negatives (X-axis) and false positives (Y-axis)

The FPR was > 50% for 156/163 HRQoL tools and > 75% for 139/163 HRQoL tools against the EQ-5D, when the prevalence of poor HRQoL was set at 50% (Fig. 3). Using the same prevalence of poor HRQoL, 156/163 tools had a FNR of > 50% and 49/163 tools a FNR of > 75%. A prevalence of poor HRQoL of 20% increased the number of studies with a FPR of > 75% to 157/163 tools, while the FNR was < 50% for 138/163 tools. Increasing the prevalence of poor HRQoL to 80% resulted in 48/163 tools having a FPR < 50% and 162/163 tools having a FNR > 75%.

### Concordance of quality of life scores

Of the disease/condition-specific HRQoL tools identified, 141 had comparative data directly against a generic tool (Fig. 1 & Additional file 1). For rare diseases, 11 studies (including eight clinical trials) covering 10 tools provided data against the SF-36 (no comparative data against the EQ-5D was identified). One-hundred and twelve studies (including 74 clinical trials) covering 73 tools for non-rare diseases provided comparative data against the SF-36 (84 studies) and EQ-5D (65 studies), with some publications including data for both generic tools. Seventy-one studies (43 clinical trials) of 58 symptom-specific tools were available with data against the SF-36 (66 studies) and EQ-5D (31 studies), again with some studies covering both generic tools. Overall, 63% (125/198) of the publications reported results from clinical trials.

*Strong* concordance of the SF-36 results was reported for only 27% (3/11) of studies of rare disease tools, 29% (24/84) for non-rare disease tools, and 18% (12/66) for symptom-specific tools (Table 1). Results were similar for the EQ-5D against non-rare tools, with 26% (17/65) showing a *Strong* level of concordance, although this generic tool appeared to perform marginally better than the SF-36 against symptom-specific tools (29%; 9/31). A total absence of concordance was noted for 30% (25/84) and 35% (23/65) of results from studies of non-rare disease-specific tools and 36% (24/66) and 16% (5/31) for symptom-specific tools versus the SF-36 and EQ-5D, respectively.

**Table 1:** Concordance between health-related quality of life results for rare and non-rare disease and symptom-specific tools versus the SF-36 and EQ-5D

Level of Concordance	Rare Disease		Non-rare Disease		Symptom-specific	
	SF-36 (*n* = 11)	EQ-5D (*n* = 0)	SF-36 (*n* = 84)	EQ-5D (*n* = 65)	SF-36 (*n* = 66)	EQ-5D (*n* = 31)
*None*	2 (18%)	–	25 (30%)	23 (35%)	24 (36%)	5 (16%)
*Moderate*	6 (55%)	–	35 (42%)	25 (38%)	30 (45%)	17 (55%)
*Strong*	3 (27%)	–	24 (29%)	17 (26%)	12 (18%)	9 (29%)

Values represent number of studies (% of total). Rare disease: affecting < 1 in 2000 population; Non-rare diseases: ≥1 in 2000 population. Concordance: *None (No)* = significant impact on quality of life with disease/condition specific tool, but no change or the opposite impact with generic tool (or vice versa); *Moderate* = quality of life impact was scored in the same direction with both tools, but statistically significant with only one of them; *Strong* = results fully aligned (significant/non-significant impact in same direction)

For studies reporting HRQoL at multiple time points, a recorded change in HRQoL over time did not result in a reclassification of concordance for rare (0/2) or non-rare disease tools (0/34) versus the SF-36, but did for 19% (7/36) of symptom-specific tools (Table 2). For the EQ-5D, concordance was reclassified for 9% (2/23) of studies of non-rare disease tools, but not for symptom-specific tools (0/7).

**Table 2:** Change in health-related quality of life over time and impact on concordance of results between rare and non-rare and symptom-specific tools versus the SF-36 and EQ-5D

	Rare Disease		Non-rare Disease		Symptom-specific	
	SF-36 (*n* = 2)	EQ-D (*n* = 0)	SF-36 (*n* = 38)	EQ-5D (*n* = 27)	SF-36 (*n* = 42)	EQ-5D (*n* = 7)
QoL change	2 (100%)	–	34 (89%)	23 (85%)	36 (86%)	7 (100%)
*Concordance change*	*0/2 (0%)*	*–*	*0/34 (0%)*	*2/23 (9%)*	*7/36 (19%)*	*0/7 (0%)*

*n* = studies with > 1 time point. Rare disease: affecting < 1 in 2000 population; Non-rare diseases: ≥1 in 2000 population

## Discussion

An accurate measure of HRQoL is of fundamental importance when considering the clinical- and cost-effectiveness of a therapy or intervention during economic evaluations/HTAs to determine use within a healthcare system. Overestimating the impact on HRQoL could result in excessive healthcare expenditure for minimal health gain (money which could be better spent elsewhere). Conversely, underestimating the impact could cause unnecessary restrictions on use to the detriment of patients. Selection of the appropriate tool or tools to assess HRQoL is, therefore, essential. This study has found that by applying Neutral Theory, commonly used generic HRQoL tools, such as the SF-36 or EQ-5D, appear poorly aligned with disease- or condition-specific tools.

Neither the SF-36 nor the EQ-5D achieved a sensitivity and specificity for included items both > 50% against any of the 162/163 disease- or condition-specific tools included in this study. Even when using a high prevalence of poor HRQoL set at 80% (i.e. 4/5 patients with this disease/condition have a notably impacted HRQoL), less than one-third of tools had a FPR of < 50% against the generic tools (SF-36: 29% of tools; EQ-5D: 29%). The situation was worse for rare disease tools, where sensitivity ranged from 0 to 40% for the SF-36 and 0–22% for the EQ-5D. Predicated on these results, it is unsurprising, therefore, that there were low levels of concordance between HRQoL scores from the generic versus the disease/condition-specific tools (no concordance vs SF-36: 18–36% of studies; vs EQ-5D: 16–35%).

Salient limitations of this study included the necessity of having to assume a true negative fraction of 0.9, as it was not possible to know if the disease/condition-specific tool fully captures all relevant items or domains (i.e., is completely Neutral). The measure of concordance between results of the generic and disease/condition-specific tools was also necessarily crude to allow for cross comparison between multiple studies of numerous diseases/conditions. Importantly, however, a high number of studies (up to approximately one-third) reported zero concordance between generic and disease/condition tools. The use of the EQ-5D can also be considered a limitation in that this is not strictly a tool to measure HRQoL, but rather generic health status [6, 8]. The EQ-5D is, however, widely used to assess HRQoL [9, 10] and, therefore, was a valid choice for this study. It is also worthy of note that, surprisingly, a full description of 29% (65/228) of the identified tools could not be obtained, despite their publication in indexed journals. This is an unacceptably high rate; such descriptions should be a standard component of publication.

HRQoL tools generate scores on the basis of individual item measures – a construct. The concept of ‘True’ HRQoL at any given time is, therefore, important. The tools generate a value of observed HRQoL on a subject based on relevant items and lack of irrelevant items. The principle underpinning the development and use of disease- and condition-specific tools is that they are inherently more accurate than generic tools at measuring HRQoL for patients with that particular disease or condition. Thereby, closer to neutrality. However, tools have been developed for the same disease/condition that do not include all the same items and domains [13, 14]. This raises the question of what is the correct construct to ensure an accurate assessment of HRQoL for that disease/condition. Several approaches have been taken to improve the accurate assessment of HRQoL, including: the parallel use of generic and disease/condition tools [15, 16]; using mapping algorithms from disease/condition-specific tools to generic tools [10]; tailoring standard items to specific diseases/conditions [17]; and use of bolt-on items to generic questionnaires [10]. Despite these approaches, the pertinent question remains – what is acceptable accuracy for a HRQoL tool? Given the importance of having an accurate measure of the impact of a therapy or intervention on HRQoL, may be it is time for there to be rethink on how HRQoL is assessed and measured. Moreover, to consider how improvements can be made to the current widespread use of generic tools.

## Conclusions

A new theory recently proposed is that of a ‘Neutral Observer’, which provides a set of principles on which a determination of the accuracy of an observation of a given construct can be based [1]. It is theorised that the “true” value of a construct can be measured by an abstract or Neutral observer who has access to a complete list of indicators that are all relevant to the empirical measurement of a construct. This Neutral Observation thereby serves as the reference against which observations using the construct can be assessed for accuracy [1]. Adoption of such an approach in the development and assessment of HRQoL tools could improve their relevance, accuracy, and utility in economic evaluations of health interventions.

## Supplementary Information

**Additional file 1.** Details of all included and excluded tools.

**Additional file 2.** Results for inclusion of appropriate domains and items for all assessed tools vs SF-36 and EQ-5D.

## Data Availability

The datasets used and/or analysed during the current study are available from the corresponding author on reasonable request.

## Abbreviations

CI: Confidence interval
EQ-5D: EuroQol five-dimensional
FNR: False negative rate
FPR: False positive rate
HRQoL: Health-related quality of life
HTA: Health technology appraisal
NICE: National Institute for Health and Care Excellence
SF-36: Medical Outcomes Study Short Form family of measures
UCLA-PCI: University of California Los Angeles-Prostate Cancer Index
WHO: World Health Organization
