# Supplementary material for: Neutral theory: applicability and neutrality of using generic health-related quality of life tools in diseases or conditions where specific tools are available
Source: BMC Med Res Methodol. 2021 Apr 26;21:86. doi: 10.1186/s12874-021-01279-w (PMC8077839; doi:10.1186/s12874-021-01279-w)
Supplement: Supplementary file 2 — Additional file 2. Results for inclusion of appropriate domains and items for all assessed tools vs SF-36 and EQ-5D. [file 12874_2021_1279_MOESM2_ESM.pdf]

Neutral Theory: applicability and Neutrality of using generic health-related quality of life tools in diseases or conditions where specific tools are available

Ravi Jandhyala
Medialis Limited, Banbury, UK

Email: ravi@medialis.co.uk

Telephone: +44 7717 448656

Table 1.1: The table presents the potential misclassification (median proportions of false positives and negatives along with 5th and 95th percentiles) for each QOL tool as compared to SF-36. The design (total number of items, overlap) and accuracy (sensitivity, specificity) are also provided. The table presents QOL tools for rare diseases. Prevalence of poor QOL is 20%.

Tool name	Design	Accuracy	False negatives	False positives
Lupus	(43, 6)	(0.14, 0.12)	0.64 (0.33, 0.91)	0.96 (0.88, 1.00)
Kids*T IPT ools*	(28, 3)	(0.11, 0.09)	0.73 (0.40, 1.00)	1.00 (0.90, 1.00)
IQol32	(31, 4)	(0.13, 0.09)	0.71 (0.40, 1.00)	0.96 (0.89, 1.00)
FBI	(25, 1)	(0.04, 0.06)	0.83 (0.50, 1.00)	1.00 (0.95, 1.00)
ASQoL	(18, 1)	(0.06, 0.06)	0.83 (0.50, 1.00)	1.00 (0.94, 1.00)
PKU-QoL	(65, 7)	(0.11, 0.18)	0.56 (0.27, 0.83)	1.00 (0.89, 1.00)
CFQ-R	(50, 20)	(0.40, 0.25)	0.36 (0.11, 0.67)	0.89 (0.75, 1.00)
EORTC QLQ-OV28	(19, 0)	(0.00, 0.05)	0.83 (0.55, 1.00)	1.00 (1.00, 1.00)
EORTC QLQ-PAN26	(26, 2)	(0.08, 0.08)	0.75 (0.43, 1.00)	1.00 (0.91, 1.00)
PANQOL	(26, 3)	(0.12, 0.09)	0.73 (0.40, 1.00)	0.96 (0.90, 1.00)
ITP-PAQ	(44, 6)	(0.14, 0.12)	0.67 (0.33, 0.91)	0.96 (0.88, 1.00)
AGDHA	(25, 4)	(0.16, 0.06)	0.80 (0.50, 1.00)	0.96 (0.88, 1.00)
The Health assessment questionnaire disability index (HAQ-DI)	(126, 6)	(0.05, 0.31)	0.43 (0.20, 0.67)	1.00 (0.92, 1.00)
Toronto Extremity Salvage Score (TESS)	(32, 5)	(0.16, 0.09)	0.71 (0.38, 1.00)	0.96 (0.88, 1.00)
UCLA-GIT	(34, 2)	(0.06, 0.08)	0.75 (0.43, 1.00)	1.00 (0.93, 1.00)
Myasthenia Gravis Questionnaire (MGQ)	(15, 2)	(0.13, 0.06)	0.80 (0.50, 1.00)	0.96 (0.89, 1.00)
ALSAQ-40	(40, 3)	(0.07, 0.11)	0.67 (0.38, 1.00)	1.00 (0.91, 1.00)

Table 1.2: The table presents the potential misclassification (median proportions of false positives and negatives along with 5th and 95th percentiles) for each QOL tool as compared to SF-36. The design (total number of items, overlap) and accuracy (sensitivity, specificity) are also provided. The table presents QOL tools for rare diseases. Prevalence of poor QOL is 50%.

Tool name	Design	Accuracy	False negatives	False positives
Lupus	(43, 6)	(0.14, 0.12)	0.88 (0.71, 1.00)	0.88 (0.71, 1.00)
Kids$T IPT ools$	(28, 3)	(0.11, 0.09)	0.93 (0.77, 1.00)	0.91 (0.75, 1.00)
IQol32	(31, 4)	(0.13, 0.09)	0.92 (0.76, 1.00)	0.88 (0.72, 1.00)
FBI	(25, 1)	(0.04, 0.06)	0.94 (0.83, 1.00)	1.00 (0.86, 1.00)
ASQoL	(18, 1)	(0.06, 0.06)	0.94 (0.82, 1.00)	0.94 (0.83, 1.00)
PKU-QoL	(65, 7)	(0.11, 0.18)	0.84 (0.67, 1.00)	0.90 (0.73, 1.00)
CFQ-R	(50, 20)	(0.40, 0.25)	0.71 (0.50, 0.92)	0.67 (0.46, 0.84)
EORTC QLQ-OV28	(19, 0)	(0.00, 0.05)	0.94 (0.84, 1.00)	1.00 (1.00, 1.00)
EORTC QLQ-PAN26	(26, 2)	(0.08, 0.08)	0.93 (0.79, 1.00)	0.93 (0.79, 1.00)
PANQOL	(26, 3)	(0.12, 0.09)	0.92 (0.77, 1.00)	0.89 (0.74, 1.00)
ITP-PAQ	(44, 6)	(0.14, 0.12)	0.88 (0.71, 1.00)	0.88 (0.71, 1.00)
AGDHA	(25, 4)	(0.16, 0.06)	0.93 (0.80, 1.00)	0.87 (0.70, 1.00)
The Health assessment questionnaire disability index (HAQ-DI)	(126, 6)	(0.05, 0.31)	0.76 (0.58, 0.91)	0.94 (0.79, 1.00)
Toronto Extremity Salvage Score (TESS)	(32, 5)	(0.16, 0.09)	0.92 (0.75, 1.00)	0.87 (0.69, 1.00)
UCLA-GIT	(34, 2)	(0.06, 0.08)	0.93 (0.79, 1.00)	0.94 (0.82, 1.00)
Myasthenia Gravis Questionnaire (MGQ)	(15, 2)	(0.13, 0.06)	0.94 (0.81, 1.00)	0.88 (0.73, 1.00)
ALSAQ-40	(40, 3)	(0.07, 0.11)	0.90 (0.75, 1.00)	0.93 (0.79, 1.00)

Table 1.3: The table presents the potential misclassification (median proportions of false positives and negatives along with 5th and 95th percentiles) for each QOL tool as compared to SF-36. The design (total number of items, overlap) and accuracy (sensitivity, specificity) are also provided. The table presents QOL tools for rare diseases. Prevalence of poor QOL is 80%.

Tool name	Design	Accuracy	False negatives	False positives
Lupus	(43, 6)	(0.14, 0.12)	0.96 (0.89, 1.00)	0.62 (0.30, 0.88)
Kids*T IP Tools*	(28, 3)	(0.11, 0.09)	1.00 (0.91, 1.00)	0.67 (0.38, 1.00)
IQol32	(31, 4)	(0.13, 0.09)	1.00 (0.91, 1.00)	0.64 (0.33, 0.90)
FBI	(25, 1)	(0.04, 0.06)	1.00 (0.95, 1.00)	0.86 (0.57, 1.00)
ASQoL	(18, 1)	(0.06, 0.06)	1.00 (0.95, 1.00)	0.83 (0.50, 1.00)
PKU-QoL	(65, 7)	(0.11, 0.18)	0.96 (0.87, 1.00)	0.67 (0.33, 1.00)
CFQ-R	(50, 20)	(0.40, 0.25)	0.92 (0.77, 1.00)	0.31 (0.11, 0.54)
EORTC QLQ-OV28	(19, 0)	(0.00, 0.05)	1.00 (0.95, 1.00)	1.00 (1.00, 1.00)
EORTC QLQ-PAN26	(26, 2)	(0.08, 0.08)	1.00 (0.91, 1.00)	0.75 (0.43, 1.00)
PANQOL	(26, 3)	(0.12, 0.09)	1.00 (0.91, 1.00)	0.67 (0.33, 1.00)
ITP-PAQ	(44, 6)	(0.14, 0.12)	0.96 (0.89, 1.00)	0.62 (0.33, 0.89)
AGDHA	(25, 4)	(0.16, 0.06)	1.00 (0.93, 1.00)	0.60 (0.30, 0.86)
The Health assessment questionnaire disability index (HAQ-DI)	(126, 6)	(0.05, 0.31)	0.93 (0.83, 1.00)	0.80 (0.43, 1.00)
Toronto Extremity Salvage Score (TESS)	(32, 5)	(0.16, 0.09)	1.00 (0.90, 1.00)	0.60 (0.29, 0.86)
UCLA-GIT	(34, 2)	(0.06, 0.08)	1.00 (0.92, 1.00)	0.80 (0.50, 1.00)
Myasthenia Gravis Questionnaire (MGQ)	(15, 2)	(0.13, 0.06)	1.00 (0.94, 1.00)	0.64 (0.33, 0.90)
ALSAQ-40	(40, 3)	(0.07, 0.11)	1.00 (0.90, 1.00)	0.75 (0.43, 1.00)

Table 1.5: The table presents the potential misclassification (median proportions of false positives and negatives along with 5th and 95th percentiles) for each QOL tool as compared to SF-36. The design (total number of items, overlap), accuracy (sensitivity, specificity) are also provided. The table presents disease-specific QOL tools with 10 highest and 10 lowest accuracy (sensitivity + specificity). Prevalence of poor QOL is 20%.

Tool name	Design	Accuracy	False negatives	False positives
KDQOL-SF	(82, 35)	(0.43, 1.00)	0.36 (0.20, 0.53)	0.00 (0.00, 0.00)
QUALIOST	(30, 30)	(1.00, 0.38)	0.00 (0.00, 0.00)	0.38 (0.23, 0.56)
CRQ-SAS 17	(17, 9)	(0.53, 0.07)	0.88 (0.62, 1.00)	0.64 (0.46, 0.81)
Angina Pectoris Quality of Life Questionnaire (APQLQ)	(22, 11)	(0.50, 0.08)	0.88 (0.64, 1.00)	0.65 (0.48, 0.82)
K-BILD	(10, 5)	(0.50, 0.03)	1.00 (0.75, 1.00)	0.67 (0.48, 0.83)
Clinical COPD	(14, 6)	(0.43, 0.03)	1.00 (0.80, 1.00)	0.70 (0.52, 0.86)
EORTC QLQ-LC13	(14, 6)	(0.43, 0.03)	1.00 (0.80, 1.00)	0.70 (0.52, 0.86)
Inflammatory Bowel Disease Questionnaire (IBDQ)	(32, 11)	(0.34, 0.11)	0.87 (0.67, 1.00)	0.73 (0.54, 0.89)
Chronic Respiratory Questionnaire (CRQ)	(46, 12)	(0.26, 0.18)	0.81 (0.62, 1.00)	0.76 (0.57, 0.93)
Quality of Life in Epilepsy Inventory (QOLIE-31P)	(38, 11)	(0.29, 0.14)	0.85 (0.64, 1.00)	0.75 (0.56, 0.92)
Epilepsy-related Fears in Parents Questionnaire (EFPQ)	(17, 0)	(0.00, 0.05)	0.94 (0.84, 1.00)	1.00 (1.00, 1.00)
Stigma (Epilepsy)	(24, 0)	(0.00, 0.05)	0.94 (0.84, 1.00)	1.00 (1.00, 1.00)
MADRS-S	(10, 0)	(0.00, 0.03)	1.00 (0.89, 1.00)	1.00 (1.00, 1.00)
ICSmaleSF	(14, 0)	(0.00, 0.03)	1.00 (0.89, 1.00)	1.00 (1.00, 1.00)
International Prostate Symptom Score (I-PSS)	(8, 0)	(0.00, 0.03)	1.00 (0.89, 1.00)	1.00 (1.00, 1.00)
The Glaucoma Symptom Scale Questionanire (GSSQ)	(10, 0)	(0.00, 0.03)	1.00 (0.89, 1.00)	1.00 (1.00, 1.00)
Chronic Sinusitis Survey (CSS)	(6, 0)	(0.00, 0.03)	1.00 (0.89, 1.00)	1.00 (1.00, 1.00)
WHO-DTSQ	(8, 0)	(0.00, 0.03)	1.00 (0.89, 1.00)	1.00 (1.00, 1.00)
BPH Index (Bothersome Index)	(8, 0)	(0.00, 0.03)	1.00 (0.89, 1.00)	1.00 (1.00, 1.00)
ICIQ-UI	(3, 0)	(0.00, 0.00)	1.00 (1.00, 1.00)	1.00 (1.00, 1.00)

Table 1.6: The table presents the potential misclassification (median proportions of false positives and negatives along with 5th and 95th percentiles) for each QOL tool as compared to SF-36. The design (total number of items, overlap), accuracy (sensitivity, specificity) are also provided. The table presents disease-specific QOL tools with 10 highest and 10 lowest accuracy (sensitivity + specificity). Prevalence of poor QOL is 20%.

Tool name	Design	Accuracy	False negatives	False positives
KDQOL-SF	(82, 35)	(0.43, 1.00)	0.70 (0.52, 0.86)	0.00 (0.00, 0.00)
QUALIOST	(30, 30)	(1.00, 0.38)	0.00 (0.00, 0.00)	0.14 (0.04, 0.25)
CRQ-SAS 17	(17, 9)	(0.53, 0.07)	1.00 (0.86, 1.00)	0.30 (0.12, 0.50)
Angina Pectoris Quality of Life Questionnaire (APQLQ)	(22, 11)	(0.50, 0.08)	1.00 (0.86, 1.00)	0.31 (0.13, 0.50)
K-BILD	(10, 5)	(0.50, 0.03)	1.00 (0.91, 1.00)	0.32 (0.14, 0.52)
Clinical COPD	(14, 6)	(0.43, 0.03)	1.00 (0.92, 1.00)	0.36 (0.16, 0.56)
EORTC QLQ-LC13	(14, 6)	(0.43, 0.03)	1.00 (0.92, 1.00)	0.36 (0.16, 0.56)
Inflammatory Bowel Disease Questionnaire (IBDQ)	(32, 11)	(0.34, 0.11)	1.00 (0.87, 1.00)	0.38 (0.17, 0.62)
Chronic Respiratory Questionnaire (CRQ)	(46, 12)	(0.26, 0.18)	0.95 (0.84, 1.00)	0.44 (0.18, 0.70)
Quality of Life in Epilepsy Inventory (QOLIE-31P)	(38, 11)	(0.29, 0.14)	0.95 (0.86, 1.00)	0.42 (0.18, 0.67)
Epilepsy-related Fears in Parents Questionnaire (EFPQ)	(17, 0)	(0.00, 0.05)	1.00 (0.95, 1.00)	1.00 (1.00, 1.00)
Stigma (Epilepsy)	(24, 0)	(0.00, 0.05)	1.00 (0.95, 1.00)	1.00 (1.00, 1.00)
MADRS-S	(10, 0)	(0.00, 0.03)	1.00 (0.96, 1.00)	1.00 (1.00, 1.00)
ICSmaleSF	(14, 0)	(0.00, 0.03)	1.00 (0.96, 1.00)	1.00 (1.00, 1.00)
International Prostate Symptom Score (I-PSS)	(8, 0)	(0.00, 0.03)	1.00 (0.96, 1.00)	1.00 (1.00, 1.00)
The Glaucoma Symptom Scale Questionanire (GSSQ)	(10, 0)	(0.00, 0.03)	1.00 (0.96, 1.00)	1.00 (1.00, 1.00)
Chronic Sinusitis Survey (CSS)	(6, 0)	(0.00, 0.03)	1.00 (0.96, 1.00)	1.00 (1.00, 1.00)
WHO-DTSQ	(8, 0)	(0.00, 0.03)	1.00 (0.96, 1.00)	1.00 (1.00, 1.00)
BPH Index (Bothersome Index)	(8, 0)	(0.00, 0.03)	1.00 (0.96, 1.00)	1.00 (1.00, 1.00)
ICIQ-UI	(3, 0)	(0.00, 0.00)	1.00 (1.00, 1.00)	1.00 (1.00, 1.00)

Table 1.7: The table presents the potential misclassification (median proportions of false positives and negatives along with 5th and 95th percentiles) for each QOL tool as compared to SF-36. The design (total number of items, overlap) and accuracy (sensitivity, specificity) are also provided. The table presents symptom-specific QOL tools with 10 highest and 10 lowest accuracy (sensitivity + specificity). Prevalence of poor QOL is 20%.

Tool name	Design	Accuracy	False negatives	False positives
SAQLI Calgary Sleep Apnea Quality of Life Index	(19, 10)	(0.53, 0.07)	0.62 (0.17, 1.00)	0.88 (0.76, 0.96)
FACT-Leu	(5, 3)	(0.60, 0.00)	1.00 (1.00, 1.00)	0.87 (0.76, 0.96)
Fact$_G$	(15, 6)	(0.40, 0.06)	0.71 (0.33, 1.00)	0.91 (0.80, 1.00)
SCQOLIT	(29, 10)	(0.34, 0.11)	0.60 (0.25, 1.00)	0.92 (0.81, 1.00)
Asthma Quality of Life Questionnaire (AQLQ)	(51, 13)	(0.25, 0.19)	0.50 (0.20, 0.80)	0.94 (0.82, 1.00)
Clinical COPD	(28, 9)	(0.32, 0.10)	0.62 (0.25, 1.00)	0.92 (0.81, 1.00)
Angina Pectoris Quality of Life Questionnaire (APQLQ)	(17, 6)	(0.35, 0.06)	0.75 (0.33, 1.00)	0.92 (0.81, 1.00)
Quality of Life in Epilepsy Inventory (QOLIE-31P)	(13, 5)	(0.38, 0.03)	0.86 (0.50, 1.00)	0.92 (0.81, 1.00)
SNOT-22	(13, 5)	(0.38, 0.03)	0.86 (0.50, 1.00)	0.92 (0.81, 1.00)
EORTC$_Q LQ_C$ 30	(55, 11)	(0.20, 0.20)	0.50 (0.22, 0.78)	0.95 (0.84, 1.00)
DDS (Diabetes Distress Scale)	(18, 0)	(0.00, 0.05)	0.83 (0.55, 1.00)	1.00 (1.00, 1.00)
ICIQ-UI	(14, 0)	(0.00, 0.03)	1.00 (0.67, 1.00)	1.00 (1.00, 1.00)
Assessment of SpondyloArthritis International Society (ASAS)	(12, 0)	(0.00, 0.03)	1.00 (0.67, 1.00)	1.00 (1.00, 1.00)
Rhinitis QOL Questionnaire (RQLQ)	(9, 0)	(0.00, 0.03)	1.00 (0.67, 1.00)	1.00 (1.00, 1.00)
Fibromyalgia impact questionnaire (FIQ)	(6, 0)	(0.00, 0.03)	1.00 (0.67, 1.00)	1.00 (1.00, 1.00)
Rhinusitis Disability Index (RSDI)	(10, 0)	(0.00, 0.03)	1.00 (0.67, 1.00)	1.00 (1.00, 1.00)
1 (MSQ)	(11, 0)	(0.00, 0.03)	1.00 (0.67, 1.00)	1.00 (1.00, 1.00)
PDQ-8	(3, 0)	(0.00, 0.00)	1.00 (1.00, 1.00)	1.00 (1.00, 1.00)
Beck Depression Inventory-II (BDI-II)	(4, 0)	(0.00, 0.00)	1.00 (1.00, 1.00)	1.00 (1.00, 1.00)
WHO-DTSQ	(5, 0)	(0.00, 0.00)	1.00 (1.00, 1.00)	1.00 (1.00, 1.00)

Table 1.8: The table presents the potential misclassification (median proportions of false positives and negatives along with 5th and 95th percentiles) for each QOL tool as compared to SF-36. The design (total number of items, overlap) and accuracy (sensitivity, specificity) are also provided. The table presents symptom-specific QOL tools with 10 highest and 10 lowest accuracy (sensitivity + specificity). Prevalence of poor QOL is 20%.

Tool name	Design	Accuracy	False negatives	False positives
SAQLI Calgary Sleep Apnea Quality of Life Index	(19, 10)	(0.53, 0.07)	0.88 (0.62, 1.00)	0.64 (0.46, 0.81)
FACT-Leu	(5, 3)	(0.60, 0.00)	1.00 (1.00, 1.00)	0.62 (0.46, 0.79)
Fact$_G$	(15, 6)	(0.40, 0.06)	0.91 (0.71, 1.00)	0.71 (0.53, 0.86)
SCQOLIT	(29, 10)	(0.34, 0.11)	0.88 (0.67, 1.00)	0.73 (0.54, 0.89)
Asthma Quality of Life Questionnaire (AQLQ)	(51, 13)	(0.25, 0.19)	0.80 (0.60, 1.00)	0.77 (0.57, 0.93)
Clinical COPD	(28, 9)	(0.32, 0.10)	0.88 (0.67, 1.00)	0.74 (0.56, 0.90)
Angina Pectoris Quality of Life Questionnaire (APQLQ)	(17, 6)	(0.35, 0.06)	0.92 (0.75, 1.00)	0.73 (0.55, 0.89)
Quality of Life in Epilepsy Inventory (QOLIE-31P)	(13, 5)	(0.38, 0.03)	1.00 (0.80, 1.00)	0.72 (0.55, 0.88)
SNOT-22	(13, 5)	(0.38, 0.03)	1.00 (0.80, 1.00)	0.72 (0.55, 0.88)
EORTC$QLQC$ 30	(55, 11)	(0.20, 0.20)	0.80 (0.62, 0.95)	0.81 (0.62, 0.95)
DDS (Diabetes Distress Scale)	(18, 0)	(0.00, 0.05)	0.94 (0.84, 1.00)	1.00 (1.00, 1.00)
ICIQ-UI	(14, 0)	(0.00, 0.03)	1.00 (0.89, 1.00)	1.00 (1.00, 1.00)
Assessment of SpondyloArthritis International Society (ASAS)	(12, 0)	(0.00, 0.03)	1.00 (0.89, 1.00)	1.00 (1.00, 1.00)
Rhinitis QOL Questionnaire (RQLQ)	(9, 0)	(0.00, 0.03)	1.00 (0.89, 1.00)	1.00 (1.00, 1.00)
Fibromyalgia impact questionnaire (FIQ)	(6, 0)	(0.00, 0.03)	1.00 (0.89, 1.00)	1.00 (1.00, 1.00)
Rhinusitis Disability Index (RSDI)	(10, 0)	(0.00, 0.03)	1.00 (0.89, 1.00)	1.00 (1.00, 1.00)
1 (MSQ)	(11, 0)	(0.00, 0.03)	1.00 (0.89, 1.00)	1.00 (1.00, 1.00)
PDQ-8	(3, 0)	(0.00, 0.00)	1.00 (1.00, 1.00)	1.00 (1.00, 1.00)
Beck Depression Inventory-II (BDI-II)	(4, 0)	(0.00, 0.00)	1.00 (1.00, 1.00)	1.00 (1.00, 1.00)
WHO-DTSQ	(5, 0)	(0.00, 0.00)	1.00 (1.00, 1.00)	1.00 (1.00, 1.00)

Table 1.9: The table presents the potential misclassification (median proportions of false positives and negatives along with 5th and 95th percentiles) for each QOL tool as compared to SF-36. The design (total number of items, overlap) and accuracy (sensitivity, specificity) are also provided. The table presents symptom-specific QOL tools with 10 highest and 10 lowest accuracy (sensitivity + specificity). Prevalence of poor QOL is 20%.

Tool name	Design	Accuracy	False negatives	False positives
SAQLI Calgary Sleep Apnea Quality of Life Index	(19, 10)	(0.53, 0.07)	1.00 (0.86, 1.00)	0.30 (0.12, 0.50)
FACT-Leu	(5, 3)	(0.60, 0.00)	1.00 (1.00, 1.00)	0.29 (0.13, 0.47)
Fact$_G$	(15, 6)	(0.40, 0.06)	1.00 (0.89, 1.00)	0.36 (0.17, 0.58)
SCQOLIT	(29, 10)	(0.34, 0.11)	1.00 (0.87, 1.00)	0.38 (0.17, 0.62)
Asthma Quality of Life Questionnaire (AQLQ)	(51, 13)	(0.25, 0.19)	0.95 (0.84, 1.00)	0.44 (0.18, 0.70)
Clinical COPD	(28, 9)	(0.32, 0.10)	1.00 (0.88, 1.00)	0.40 (0.18, 0.64)
Angina Pectoris Quality of Life Questionnaire (APQLQ)	(17, 6)	(0.35, 0.06)	1.00 (0.89, 1.00)	0.40 (0.18, 0.62)
Quality of Life in Epilepsy Inventory (QOLIE-31P)	(13, 5)	(0.38, 0.03)	1.00 (0.92, 1.00)	0.38 (0.17, 0.60)
SNOT-22	(13, 5)	(0.38, 0.03)	1.00 (0.92, 1.00)	0.38 (0.17, 0.60)
EORTC$_Q LQ_C$ 30	(55, 11)	(0.20, 0.20)	0.95 (0.84, 1.00)	0.50 (0.22, 0.78)
DDS (Diabetes Distress Scale)	(18, 0)	(0.00, 0.05)	1.00 (0.95, 1.00)	1.00 (1.00, 1.00)
ICIQ-UI	(14, 0)	(0.00, 0.03)	1.00 (0.96, 1.00)	1.00 (1.00, 1.00)
Assessment of SpondyloArthritis International Society (ASAS)	(12, 0)	(0.00, 0.03)	1.00 (0.96, 1.00)	1.00 (1.00, 1.00)
Rhinitis QOL Questionnaire (RQLQ)	(9, 0)	(0.00, 0.03)	1.00 (0.96, 1.00)	1.00 (1.00, 1.00)
Fibromyalgia impact questionnaire (FIQ)	(6, 0)	(0.00, 0.03)	1.00 (0.96, 1.00)	1.00 (1.00, 1.00)
Rhinusitis Disability Index (RSDI)	(10, 0)	(0.00, 0.03)	1.00 (0.96, 1.00)	1.00 (1.00, 1.00)
1 (MSQ)	(11, 0)	(0.00, 0.03)	1.00 (0.96, 1.00)	1.00 (1.00, 1.00)
PDQ-8	(3, 0)	(0.00, 0.00)	1.00 (1.00, 1.00)	1.00 (1.00, 1.00)
Beck Depression Inventory-II (BDI-II)	(4, 0)	(0.00, 0.00)	1.00 (1.00, 1.00)	1.00 (1.00, 1.00)
WHO-DTSQ	(5, 0)	(0.00, 0.00)	1.00 (1.00, 1.00)	1.00 (1.00, 1.00)

Table 2.1: The table presents the potential misclassification (median proportions of false positives and negatives along with 5th and 95th percentiles) for each QOL tool as compared to EQ-5D. The design (total number of items, overlap) and accuracy (sensitivity, specificity) are also provided. The table presents QOL tools for rare diseases. Prevalence of poor QOL is 20%.

Tool name	Design	Accuracy	False negatives	False positives	Clusters
Lupus	(43, 4)	(0.09, 0.80)	0.22 (0.08, 0.36)	1.00 (0.60, 1.00)	2
Kids*T IP Tools*	(28, 2)	(0.07, 0.50)	0.31 (0.13, 0.50)	1.00 (0.86, 1.00)	2
IQol32	(31, 3)	(0.10, 0.60)	0.27 (0.11, 0.44)	1.00 (0.80, 1.00)	1
FBI	(25, 1)	(0.04, 0.33)	0.42 (0.20, 0.65)	1.00 (0.92, 1.00)	1
ASQoL	(18, 4)	(0.22, 0.67)	0.22 (0.08, 0.38)	0.88 (0.62, 1.00)	0
PKU-QoL	(65, 3)	(0.05, 0.75)	0.24 (0.09, 0.39)	1.00 (0.75, 1.00)	3
CFQ-R	(50, 2)	(0.04, 0.62)	0.27 (0.11, 0.44)	1.00 (0.86, 1.00)	0
EORTC QLQ-OV28	(19, 1)	(0.05, 0.33)	0.41 (0.19, 0.64)	1.00 (0.92, 1.00)	4
EORTC QLQ-PAN26	(26, 2)	(0.08, 0.50)	0.31 (0.13, 0.50)	1.00 (0.86, 1.00)	0
PANQOL	(26, 2)	(0.08, 0.50)	0.31 (0.13, 0.50)	1.00 (0.86, 1.00)	0
ITP-PAQ	(44, 2)	(0.05, 0.57)	0.29 (0.12, 0.47)	1.00 (0.88, 1.00)	0
AGDHA	(25, 0)	(0.00, 0.29)	0.46 (0.23, 0.70)	1.00 (1.00, 1.00)	2
The Health assessment questionnaire disability index (HAQ-DI)	(126, 3)	(0.02, 0.87)	0.21 (0.08, 0.36)	1.00 (0.67, 1.00)	0
Toronto Extremity Salvage Score (TESS)	(32, 2)	(0.06, 0.50)	0.31 (0.13, 0.50)	1.00 (0.87, 1.00)	1
UCLA-GIT	(34, 0)	(0.00, 0.38)	0.40 (0.19, 0.62)	1.00 (1.00, 1.00)	1
Myasthenia Gravis Questionnaire (MGQ)	(15, 3)	(0.20, 0.50)	0.28 (0.11, 0.47)	0.92 (0.75, 1.00)	2
ALSAQ-40	(40, 5)	(0.12, 1.00)	0.17 (0.07, 0.30)	0.00 (0.00, 0.00)	2

Table 2.2: The table presents the potential misclassification (median proportions of false positives and negatives along with 5th and 95th percentiles) for each QOL tool as compared to EQ-5D. The design (total number of items, overlap) and accuracy (sensitivity, specificity) are also provided. The table presents QOL tools for rare diseases. Prevalence of poor QOL is 50%.

Tool name	Design	Accuracy	False negatives	False positives	Clusters
Lupus	(43, 4)	(0.09, 0.80)	0.54 (0.37, 0.69)	0.67 (0.25, 1.00)	2
Kids*T IPT ools*	(28, 2)	(0.07, 0.50)	0.65 (0.48, 0.82)	0.89 (0.67, 1.00)	2
IQol32	(31, 3)	(0.10, 0.60)	0.60 (0.43, 0.76)	0.83 (0.50, 1.00)	1
FBI	(25, 1)	(0.04, 0.33)	0.75 (0.57, 0.90)	1.00 (0.80, 1.00)	1
ASQoL	(18, 4)	(0.22, 0.67)	0.54 (0.36, 0.71)	0.60 (0.29, 0.88)	0
PKU-QoL	(65, 3)	(0.05, 0.75)	0.56 (0.39, 0.72)	0.89 (0.50, 1.00)	3
CFQ-R	(50, 2)	(0.04, 0.62)	0.61 (0.43, 0.77)	1.00 (0.67, 1.00)	0
EORTC QLQ-OV28	(19, 1)	(0.05, 0.33)	0.75 (0.56, 0.90)	0.93 (0.78, 1.00)	4
EORTC QLQ-PAN26	(26, 2)	(0.08, 0.50)	0.65 (0.47, 0.82)	0.88 (0.64, 1.00)	0
PANQOL	(26, 2)	(0.08, 0.50)	0.65 (0.47, 0.82)	0.88 (0.64, 1.00)	0
ITP-PAQ	(44, 2)	(0.05, 0.57)	0.62 (0.45, 0.79)	1.00 (0.67, 1.00)	0
AGDHA	(25, 0)	(0.00, 0.29)	0.78 (0.61, 0.94)	1.00 (1.00, 1.00)	2
The Health assessment questionnaire disability index (HAQ-DI)	(126, 3)	(0.02, 0.87)	0.53 (0.37, 0.68)	1.00 (0.33, 1.00)	0
Toronto Extremity Salvage Score (TESS)	(32, 2)	(0.06, 0.50)	0.65 (0.48, 0.82)	0.90 (0.67, 1.00)	1
UCLA-GIT	(34, 0)	(0.00, 0.38)	0.73 (0.56, 0.89)	1.00 (1.00, 1.00)	1
Myasthenia Gravis Questionnaire (MGQ)	(15, 3)	(0.20, 0.50)	0.62 (0.43, 0.80)	0.73 (0.45, 0.93)	2
ALSAQ-40	(40, 5)	(0.12, 1.00)	0.46 (0.31, 0.62)	0.00 (0.00, 0.00)	2

Table 2.3: The table presents the potential misclassification (median proportions of false positives and negatives along with 5th and 95th percentiles) for each QOL tool as compared to EQ-5D. The design (total number of items, overlap) and accuracy (sensitivity, specificity) are also provided. The table presents QOL tools for rare diseases. Prevalence of poor QOL is 80%.

Tool name	Design	Accuracy	False negatives	False positives	Clusters
Lupus	(43, 4)	(0.09, 0.80)	0.82 (0.68, 0.93)	0.33 (0.00, 1.00)	2
Kids*T IPT ools*	(28, 2)	(0.07, 0.50)	0.88 (0.77, 0.96)	0.67 (0.20, 1.00)	2
IQol32	(31, 3)	(0.10, 0.60)	0.86 (0.73, 0.96)	0.50 (0.00, 1.00)	1
FBI	(25, 1)	(0.04, 0.33)	0.92 (0.83, 1.00)	0.83 (0.50, 1.00)	1
ASQoL	(18, 4)	(0.22, 0.67)	0.83 (0.68, 0.95)	0.25 (0.00, 0.57)	0
PKU-QoL	(65, 3)	(0.05, 0.75)	0.85 (0.71, 0.93)	0.50 (0.00, 1.00)	3
CFQ-R	(50, 2)	(0.04, 0.62)	0.86 (0.74, 0.96)	0.71 (0.00, 1.00)	0
EORTC QLQ-OV28	(19, 1)	(0.05, 0.33)	0.92 (0.82, 1.00)	0.78 (0.40, 1.00)	4
EORTC QLQ-PAN26	(26, 2)	(0.08, 0.50)	0.88 (0.77, 0.96)	0.62 (0.20, 1.00)	0
PANQOL	(26, 2)	(0.08, 0.50)	0.88 (0.77, 0.96)	0.62 (0.20, 1.00)	0
ITP-PAQ	(44, 2)	(0.05, 0.57)	0.88 (0.75, 0.96)	0.71 (0.00, 1.00)	0
AGDHA	(25, 0)	(0.00, 0.29)	0.93 (0.85, 1.00)	1.00 (1.00, 1.00)	2
The Health assessment questionnaire disability index (HAQ-DI)	(126, 3)	(0.02, 0.87)	0.83 (0.69, 0.93)	0.50 (0.00, 1.00)	0
Toronto Extremity Salvage Score (TESS)	(32, 2)	(0.06, 0.50)	0.88 (0.77, 0.97)	0.67 (0.25, 1.00)	1
UCLA-GIT	(34, 0)	(0.00, 0.38)	0.92 (0.81, 1.00)	1.00 (1.00, 1.00)	1
Myasthenia Gravis Questionnaire (MGQ)	(15, 3)	(0.20, 0.50)	0.87 (0.74, 0.96)	0.38 (0.10, 0.71)	2
ALSAQ-40	(40, 5)	(0.12, 1.00)	0.79 (0.64, 0.90)	0.00 (0.00, 0.00)	2

Table 2.4: The table presents the potential misclassification (median proportions of false positives and negatives along with 5th and 95th percentiles) for each QOL tool as compared to EQ-5D. The design (total number of items, overlap) and accuracy (sensitivity, specificity) are also provided. The table presents disease-specific QOL tools with 10 highest and 10 lowest accuracy (sensitivity + specificity). Prevalence of poor QOL is 20%.

Tool name	Design	Accuracy	False negatives	False positives	Clusters
EASi-QoL	(20, 5)	(0.25, 1.00)	0.15 (0.04, 0.28)	0.00 (0.00, 0.00)	1
QUALIOST	(30, 5)	(0.17, 1.00)	0.17 (0.07, 0.30)	0.00 (0.00, 0.00)	3
PDQ-8	(8, 4)	(0.50, 0.50)	0.19 (0.05, 0.38)	0.81 (0.62, 0.95)	3
Chronic Respiratory Questionnaire (CRQ)	(46, 4)	(0.09, 0.83)	0.21 (0.08, 0.36)	1.00 (0.50, 1.00)	0
CRQ-SAS 17	(17, 4)	(0.24, 0.67)	0.22 (0.08, 0.38)	0.86 (0.62, 1.00)	1
OAKHQoL	(43, 4)	(0.09, 0.80)	0.22 (0.08, 0.36)	1.00 (0.60, 1.00)	0
PsaQoL	(28, 4)	(0.14, 0.75)	0.22 (0.08, 0.37)	0.89 (0.60, 1.00)	2
EORTC$_{Q}LQ_{C}$ 30	(30, 4)	(0.13, 0.75)	0.22 (0.08, 0.37)	0.89 (0.62, 1.00)	2
EORTC-QLQ-C30	(22, 4)	(0.18, 0.67)	0.23 (0.09, 0.39)	0.89 (0.67, 1.00)	1
Stroke-Specific Quality of Life Scale (SS-QOL)	(78, 3)	(0.04, 0.80)	0.23 (0.09, 0.38)	1.00 (0.75, 1.00)	2
MSQ	(14, 1)	(0.07, 0.20)	0.55 (0.25, 0.80)	1.00 (0.90, 1.00)	1
ADDQol	(8, 0)	(0.00, 0.17)	0.60 (0.33, 0.86)	1.00 (1.00, 1.00)	4
ICIQ-UI	(10, 0)	(0.00, 0.17)	0.60 (0.33, 0.86)	1.00 (1.00, 1.00)	4
International Continence Society short-form male survey [ICSmaleSF]	(14, 0)	(0.00, 0.17)	0.60 (0.33, 0.86)	1.00 (1.00, 1.00)	0
MADRS-S	(8, 0)	(0.00, 0.17)	0.60 (0.33, 0.86)	1.00 (1.00, 1.00)	0
ICSmaleSF	(10, 0)	(0.00, 0.17)	0.60 (0.33, 0.86)	1.00 (1.00, 1.00)	0
Chronic Sinusitis Survey (CSS)	(6, 0)	(0.00, 0.17)	0.60 (0.33, 0.86)	1.00 (1.00, 1.00)	1
WHO-DTSQ	(8, 0)	(0.00, 0.17)	0.60 (0.33, 0.86)	1.00 (1.00, 1.00)	0
BPH Index (Bothersome Index)	(8, 0)	(0.00, 0.17)	0.60 (0.33, 0.86)	1.00 (1.00, 1.00)	1
KDQOL Cognitive Function (KDQOL-CF)	(3, 0)	(0.00, 0.00)	1.00 (1.00, 1.00)	1.00 (1.00, 1.00)	0

Table 2.5: The table presents the potential misclassification (median proportions of false positives and negatives along with 5th and 95th percentiles) for each QOL tool as compared to EQ-5D. The design (total number of items, overlap) and accuracy (sensitivity, specificity) are also provided. The table presents disease-specific QOL tools with 10 highest and 10 lowest accuracy (sensitivity + specificity). Prevalence of poor QOL is 20%.

Tool name	Design	Accuracy	False negatives	False positives	Clusters
EASi-QoL	(20, 5)	(0.25, 1.00)	0.43 (0.27, 0.59)	0.00 (0.00, 0.00)	1
QUALIOST	(30, 5)	(0.17, 1.00)	0.45 (0.30, 0.62)	0.00 (0.00, 0.00)	3
PDQ-8	(8, 4)	(0.50, 0.50)	0.50 (0.29, 0.72)	0.50 (0.29, 0.72)	3
Chronic Respiratory Questionnaire (CRQ)	(46, 4)	(0.09, 0.83)	0.52 (0.36, 0.68)	0.67 (0.00, 1.00)	0
CRQ-SAS 17	(17, 4)	(0.24, 0.67)	0.54 (0.35, 0.71)	0.60 (0.29, 0.88)	1
OAKHQoL	(43, 4)	(0.09, 0.80)	0.54 (0.37, 0.69)	0.67 (0.25, 1.00)	0
PsaQoL	(28, 4)	(0.14, 0.75)	0.54 (0.36, 0.70)	0.67 (0.25, 1.00)	2
EORTC$_{Q}LQ_{C}$ 30	(30, 4)	(0.13, 0.75)	0.54 (0.36, 0.70)	0.67 (0.25, 1.00)	2
EORTC-QLQ-C30	(22, 4)	(0.18, 0.67)	0.55 (0.38, 0.72)	0.67 (0.33, 1.00)	1
Stroke-Specific Quality of Life Scale (SS-QOL)	(78, 3)	(0.04, 0.80)	0.54 (0.38, 0.70)	1.00 (0.50, 1.00)	2
MSQ	(14, 1)	(0.07, 0.20)	0.83 (0.65, 0.95)	0.93 (0.77, 1.00)	1
ADDQol	(8, 0)	(0.00, 0.17)	0.87 (0.71, 1.00)	1.00 (1.00, 1.00)	4
ICIQ-UI	(10, 0)	(0.00, 0.17)	0.87 (0.71, 1.00)	1.00 (1.00, 1.00)	4
International Continence Society short-form male survey [ICSmaleSF]	(14, 0)	(0.00, 0.17)	0.87 (0.71, 1.00)	1.00 (1.00, 1.00)	0
MADRS-S	(8, 0)	(0.00, 0.17)	0.87 (0.71, 1.00)	1.00 (1.00, 1.00)	0
ICSmaleSF	(10, 0)	(0.00, 0.17)	0.87 (0.71, 1.00)	1.00 (1.00, 1.00)	0
Chronic Sinusitis Survey (CSS)	(6, 0)	(0.00, 0.17)	0.87 (0.71, 1.00)	1.00 (1.00, 1.00)	1
WHO-DTSQ	(8, 0)	(0.00, 0.17)	0.87 (0.71, 1.00)	1.00 (1.00, 1.00)	0
BPH Index (Bothersome Index)	(8, 0)	(0.00, 0.17)	0.87 (0.71, 1.00)	1.00 (1.00, 1.00)	1
KDQOL Cognitive Function (KDQOL-CF)	(3, 0)	(0.00, 0.00)	1.00 (1.00, 1.00)	1.00 (1.00, 1.00)	0

Table 2.6: The table presents the potential misclassification (median proportions of false positives and negatives along with 5th and 95th percentiles) for each QOL tool as compared to EQ-5D. The design (total number of items, overlap) and accuracy (sensitivity, specificity) are also provided. The table presents disease-specific QOL tools with 10 highest and 10 lowest accuracy (sensitivity + specificity). Prevalence of poor QOL is 20%.

Tool name	Design	Accuracy	False negatives	False positives	Clusters
EASi-QoL	(20, 5)	(0.25, 1.00)	0.75 (0.60, 0.89)	0.00 (0.00, 0.00)	1
QUALIOST	(30, 5)	(0.17, 1.00)	0.78 (0.63, 0.90)	0.00 (0.00, 0.00)	3
PDQ-8	(8, 4)	(0.50, 0.50)	0.81 (0.62, 0.95)	0.19 (0.05, 0.38)	3
Chronic Respiratory Questionnaire (CRQ)	(46, 4)	(0.09, 0.83)	0.82 (0.68, 0.93)	0.33 (0.00, 1.00)	0
CRQ-SAS 17	(17, 4)	(0.24, 0.67)	0.83 (0.68, 0.95)	0.25 (0.00, 0.56)	1
OAKHQoL	(43, 4)	(0.09, 0.80)	0.82 (0.68, 0.93)	0.33 (0.00, 1.00)	0
PsaQoL	(28, 4)	(0.14, 0.75)	0.83 (0.68, 0.93)	0.29 (0.00, 0.67)	2
EORTC$_{Q}LQ_{C}$ 30	(30, 4)	(0.13, 0.75)	0.83 (0.68, 0.93)	0.30 (0.00, 0.75)	2
EORTC-QLQ-C30	(22, 4)	(0.18, 0.67)	0.83 (0.69, 0.96)	0.29 (0.00, 0.67)	1
Stroke-Specific Quality of Life Scale (SS-QOL)	(78, 3)	(0.04, 0.80)	0.83 (0.70, 0.93)	0.50 (0.00, 1.00)	2
MSQ	(14, 1)	(0.07, 0.20)	0.96 (0.87, 1.00)	0.75 (0.40, 1.00)	1
ADDQol	(8, 0)	(0.00, 0.17)	0.96 (0.88, 1.00)	1.00 (1.00, 1.00)	4
ICIQ-UI	(10, 0)	(0.00, 0.17)	0.96 (0.88, 1.00)	1.00 (1.00, 1.00)	4
International Continence Society short-form male survey [ICSmaleSF]	(14, 0)	(0.00, 0.17)	0.96 (0.88, 1.00)	1.00 (1.00, 1.00)	0
MADRS-S	(8, 0)	(0.00, 0.17)	0.96 (0.88, 1.00)	1.00 (1.00, 1.00)	0
ICSmaleSF	(10, 0)	(0.00, 0.17)	0.96 (0.88, 1.00)	1.00 (1.00, 1.00)	0
Chronic Sinusitis Survey (CSS)	(6, 0)	(0.00, 0.17)	0.96 (0.88, 1.00)	1.00 (1.00, 1.00)	1
WHO-DTSQ	(8, 0)	(0.00, 0.17)	0.96 (0.88, 1.00)	1.00 (1.00, 1.00)	0
BPH Index (Bothersome Index)	(8, 0)	(0.00, 0.17)	0.96 (0.88, 1.00)	1.00 (1.00, 1.00)	1
KDQOL Cognitive Function (KDQOL-CF)	(3, 0)	(0.00, 0.00)	1.00 (1.00, 1.00)	1.00 (1.00, 1.00)	0

Table 2.7: The table presents the potential misclassifcation (median proportions of false postives and negatives along with 5th and 95th percentiles) for each QOL tool as compared to EQ-5D. The design (total number of items, overlap), accuracy (sensitivity, specificity) are also provided. The table presents symptom-specific QOL tools with 10 highest and 10 lowest accuracy (sensitivity + specificity). Prevalence of poor QOL is 20%.

Tool name	Design	Accuracy	False negatives	False positives	Cluste
PedsQl	(22, 5)	(0.23, 1.00)	0.17 (0.07, 0.28)	0.00 (0.00, 0.00)	3
Nottingham Health Profile (NHP)	(45, 5)	(0.11, 1.00)	0.17 (0.07, 0.30)	0.00 (0.00, 0.00)	1
North American Spine Society (NASS) Lumbar Spine Instrument	(65, 5)	(0.08, 1.00)	0.18 (0.07, 0.31)	0.00 (0.00, 0.00)	1
SGRQ	(55, 4)	(0.07, 0.86)	0.21 (0.08, 0.35)	1.00 (0.50, 1.00)	2
CAMPHOR	(65, 4)	(0.06, 0.86)	0.21 (0.08, 0.35)	1.00 (0.50, 1.00)	0
PROMIS	(29, 4)	(0.14, 0.75)	0.22 (0.08, 0.37)	0.89 (0.62, 1.00)	1
Severe Respiratory Insufficiency (SRI)	(49, 3)	(0.06, 0.71)	0.24 (0.10, 0.40)	1.00 (0.77, 1.00)	0
Child Health Questionnaire Parent Form 50 Questions (CHQ-PF50)	(51, 3)	(0.06, 0.71)	0.24 (0.10, 0.40)	1.00 (0.78, 1.00)	0
Thyroid-Specific Quality of Life Questionnaire (ThyroPro)	(84, 2)	(0.02, 0.73)	0.25 (0.10, 0.40)	1.00 (0.83, 1.00)	1
CHQ$_P F$ 28	(28, 3)	(0.11, 0.60)	0.26 (0.11, 0.44)	1.00 (0.78, 1.00)	1
Hospital Anxiety and Depression Scale (HADS)	(14, 1)	(0.07, 0.20)	0.55 (0.25, 0.80)	1.00 (0.90, 1.00)	0
International Consultation on Incontinence Questionnaire- Urinary Incontinence [ICIQ-UI]	(4, 1)	(0.25, 0.00)	1.00 (1.00, 1.00)	0.96 (0.85, 1.00)	2
Xerostomia Inventory (XI)	(14, 0)	(0.00, 0.17)	0.60 (0.33, 0.86)	1.00 (1.00, 1.00)	1
Ocular Surface Disease Index (OSDI)	(12, 0)	(0.00, 0.17)	0.60 (0.33, 0.86)	1.00 (1.00, 1.00)	1
General Oral Health Assessment Index (GOHAI)	(12, 0)	(0.00, 0.17)	0.60 (0.33, 0.86)	1.00 (1.00, 1.00)	0
Rosenberg Self Esteem Questionnaire (RSEQ)	(10, 0)	(0.00, 0.17)	0.60 (0.33, 0.86)	1.00 (1.00, 1.00)	1
American Urological Association (AUA) symptom score	(11, 0)	(0.00, 0.17)	0.60 (0.33, 0.86)	1.00 (1.00, 1.00)	0
WHO$_5$	(5, 0)	(0.00, 0.00)	1.00 (1.00, 1.00)	1.00 (1.00, 1.00)	0
BCSS	(3, 0)	(0.00, 0.00)	1.00 (1.00, 1.00)	1.00 (1.00, 1.00)	3
FABQ-PA	(5, 0)	(0.00, 0.00)	1.00 (1.00, 1.00)	1.00 (1.00, 1.00)	0

Table 2.8: The table presents the potential misclassifcation (median proportions of false postives and negatives along with 5th and 95th percentiles) for each QOL tool as compared to EQ-5D. The design (total number of items, overlap), accuracy (sensitivity, specificity) are also provided. The table presents symptom-specific QOL tools with 10 highest and 10 lowest accuracy (sensitivity + specificity). Prevalence of poor QOL is 20%.

Tool name	Design	Accuracy	False negatives	False positives	Cluste
PedsQl	(22, 5)	(0.23, 1.00)	0.43 (0.28, 0.60)	0.00 (0.00, 0.00)	3
Nottingham Health Profile (NHP)	(45, 5)	(0.11, 1.00)	0.47 (0.31, 0.63)	0.00 (0.00, 0.00)	1
North American Spine Society (NASS) Lumbar Spine Instrument	(65, 5)	(0.08, 1.00)	0.48 (0.32, 0.63)	0.00 (0.00, 0.00)	1
SGRQ	(55, 4)	(0.07, 0.86)	0.52 (0.36, 0.68)	0.67 (0.00, 1.00)	2
CAMPHOR	(65, 4)	(0.06, 0.86)	0.52 (0.36, 0.68)	0.75 (0.00, 1.00)	0
PROMIS	(29, 4)	(0.14, 0.75)	0.54 (0.36, 0.70)	0.67 (0.25, 1.00)	1
Severe Respiratory Insufficiency (SRI)	(49, 3)	(0.06, 0.71)	0.57 (0.40, 0.73)	0.83 (0.50, 1.00)	0
Child Health Questionnaire Parent Form 50 Questions (CHQ-PF50)	(51, 3)	(0.06, 0.71)	0.57 (0.40, 0.73)	0.86 (0.50, 1.00)	0
Thyroid-Specific Quality of Life Questionnaire (ThyroPro)	(84, 2)	(0.02, 0.73)	0.58 (0.41, 0.73)	1.00 (0.67, 1.00)	1
CHQ$_{P}F$ 28	(28, 3)	(0.11, 0.60)	0.60 (0.42, 0.76)	0.80 (0.50, 1.00)	1
Hospital Anxiety and Depression Scale (HADS)	(14, 1)	(0.07, 0.20)	0.83 (0.65, 0.95)	0.93 (0.77, 1.00)	0
International Consultation on Incontinence Questionnaire- Urinary Incontinence [ICIQ-UI]	(4, 1)	(0.25, 0.00)	1.00 (1.00, 1.00)	0.81 (0.64, 0.94)	2
Xerostomia Inventory (XI)	(14, 0)	(0.00, 0.17)	0.87 (0.71, 1.00)	1.00 (1.00, 1.00)	1
Ocular Surface Disease Index (OSDI)	(12, 0)	(0.00, 0.17)	0.87 (0.71, 1.00)	1.00 (1.00, 1.00)	1
General Oral Health Assessment Index (GOHAI)	(12, 0)	(0.00, 0.17)	0.87 (0.71, 1.00)	1.00 (1.00, 1.00)	0
Rosenberg Self Esteem Questionnaire (RSEQ)	(10, 0)	(0.00, 0.17)	0.87 (0.71, 1.00)	1.00 (1.00, 1.00)	1
American Urological Association (AUA) symptom score	(11, 0)	(0.00, 0.17)	0.87 (0.71, 1.00)	1.00 (1.00, 1.00)	0
WHO5	(5, 0)	(0.00, 0.00)	1.00 (1.00, 1.00)	1.00 (1.00, 1.00)	0
BCSS	(3, 0)	(0.00, 0.00)	1.00 (1.00, 1.00)	1.00 (1.00, 1.00)	3
FABQ-PA	(5, 0)	(0.00, 0.00)	1.00 (1.00, 1.00)	1.00 (1.00, 1.00)	0

Table 2.9: The table presents the potential misclassifcation (median proportions of false postives and negatives along with 5th and 95th percentiles) for each QOL tool as compared to EQ-5D. The design (total number of items, overlap), accuracy (sensitivity, specificity) are also provided. The table presents symptom-specific QOL tools with 10 highest and 10 lowest accuracy (sensitivity + specificity). Prevalence of poor QOL is 20%.

Tool name	Design	Accuracy	False negatives	False positives	Cluste
PedsQl	(22, 5)	(0.23, 1.00)	0.76 (0.61, 0.89)	0.00 (0.00, 0.00)	3
Nottingham Health Profile (NHP)	(45, 5)	(0.11, 1.00)	0.79 (0.64, 0.90)	0.00 (0.00, 0.00)	1
North American Spine Society (NASS) Lumbar Spine Instrument	(65, 5)	(0.08, 1.00)	0.79 (0.66, 0.90)	0.00 (0.00, 0.00)	1
SGRQ	(55, 4)	(0.07, 0.86)	0.81 (0.68, 0.93)	0.33 (0.00, 1.00)	2
CAMPHOR	(65, 4)	(0.06, 0.86)	0.82 (0.68, 0.93)	0.33 (0.00, 1.00)	0
PROMIS	(29, 4)	(0.14, 0.75)	0.83 (0.68, 0.93)	0.29 (0.00, 0.71)	1
Severe Respiratory Insufficiency (SRI)	(49, 3)	(0.06, 0.71)	0.85 (0.71, 0.96)	0.50 (0.00, 1.00)	0
Child Health Questionnaire Parent Form 50 Questions (CHQ-PF50)	(51, 3)	(0.06, 0.71)	0.85 (0.71, 0.96)	0.50 (0.00, 1.00)	0
Thyroid-Specific Quality of Life Questionnaire (ThyroPro)	(84, 2)	(0.02, 0.73)	0.85 (0.72, 0.96)	1.00 (0.00, 1.00)	1
CHQ$_P F$ 28	(28, 3)	(0.11, 0.60)	0.86 (0.73, 0.96)	0.50 (0.00, 1.00)	1
Hospital Anxiety and Depression Scale (HADS)	(14, 1)	(0.07, 0.20)	0.96 (0.87, 1.00)	0.75 (0.40, 1.00)	0
International Consultation on Incontinence Questionnaire- Urinary Incontinence [ICIQ-UI]	(4, 1)	(0.25, 0.00)	1.00 (1.00, 1.00)	0.50 (0.25, 0.75)	2
Xerostomia Inventory (XI)	(14, 0)	(0.00, 0.17)	0.96 (0.88, 1.00)	1.00 (1.00, 1.00)	1
Ocular Surface Disease Index (OSDI)	(12, 0)	(0.00, 0.17)	0.96 (0.88, 1.00)	1.00 (1.00, 1.00)	1
General Oral Health Assessment Index (GOHAI)	(12, 0)	(0.00, 0.17)	0.96 (0.88, 1.00)	1.00 (1.00, 1.00)	0
Rosenberg Self Esteem Questionnaire (RSEQ)	(10, 0)	(0.00, 0.17)	0.96 (0.88, 1.00)	1.00 (1.00, 1.00)	1
American Urological Association (AUA) symptom score	(11, 0)	(0.00, 0.17)	0.96 (0.88, 1.00)	1.00 (1.00, 1.00)	0
WHO$_5$	(5, 0)	(0.00, 0.00)	1.00 (1.00, 1.00)	1.00 (1.00, 1.00)	0
BCSS	(3, 0)	(0.00, 0.00)	1.00 (1.00, 1.00)	1.00 (1.00, 1.00)	3
FABQ-PA	(5, 0)	(0.00, 0.00)	1.00 (1.00, 1.00)	1.00 (1.00, 1.00)	0

Table 3.1: QOL Tools and reviewed scientific papers

	Rare-disease	Disease-specific	Subject-specific
Number of tools	10	73	58
Number of papers	15	112	71
Number of clinical trials	8	74	43

Table 3.2: Comparison against general QOL Tools and reviewed scientific papers

	**Rare-disease**		**Disease-specific**		**Subject-specific**	
	SF-36	EQ5D	SF-36	EQ5D	SF-36	EQ5D
Concordance 1	2 (18.18)	0 (NaN)	25 (29.76)	23 (35.38)	24 (36.36)	5 (16.13)
Concordance 2	6 (54.55)	0 (NaN)	35 (41.67)	25 (38.46)	30 (45.45)	17 (54.84)
Concordance 3	3 (27.27)	0 (NaN)	24 (28.57)	17 (26.15)	12 (18.18)	9 (29.03)
Timepoints >1	2	0	38	27	42	7
QOL change	2	0	34	23	36	7
Concordance change	0	0	0	2	7	0
